# Electrically Conductive Nanocarbon/Elastomer Composite Inks for Flexible and Wearable Strain Sensing

**DOI:** 10.1002/smll.202506844

**Published:** 2025-11-14

**Authors:** Siva Sankar Nemala, Bruno Bernardino, Rui M. R. Pinto, Vicente Lopes, Pedro Alpuim, Ihsan Çaha, Edoardo Sotgiu, Marián A. Gómez‐Fatou, Juan Francisco Vega, Horacio Javier Salavagione, Andrea Capasso

**Affiliations:** ^1^ International Iberian Nanotechnology Laboratory Braga 4715‐330 Portugal; ^2^ Centro de Física das Universidades do Minho e Porto Universidade do Minho Braga 4710‐057 Portugal; ^3^ Instituto de Ciencia y Tecnología de Polímeros (ICTP) CSIC Departamento de Física de Polímeros Elastómeros y Aplicaciones Energéticas C/Juan de la Cierva 3 Madrid 28006 Spain; ^4^ Instituto de Estructura de la Materia (IEM) CSIC Departamento de Física Macromolecular BIOPHYM Serrano 113bis Madrid 28006 Spain

**Keywords:** elastomers, e‐textiles, hybrid nanomaterials, printable inks, solution processing

## Abstract

Flexible strain sensors are essential components of wearable devices for health monitoring, motion tracking, human‐machine interaction, and rehabilitation. Here, we report an eco‐friendly, all‐carbon conductive ink composed of carbon nano‐onions (CNOs) and carbon nanotubes (CNTs) dispersed in a poly(styrene‐ethylene‐butylene‐styrene) elastomeric matrix. The ink is formulated using biomass‐derived 2‐methyltetrahydrofuran to ensure environmental compatibility. The combination of 0D CNOs and 1D CNTs provides high electrical conductivity, mechanical robustness, and tunable viscosity. Compressive strain sensors prepared by dip coating polyurethane sponges show a modulus of ≈460 kPa, a gauge factor of ≈1.1, and electrical hysteresis of 11.3% under 75% compression. Integrated into a football, the sensors detect contact, rotation, and rebound. Tensile strain sensors made by blade coating on stretchable textiles achieve gauge factors of 10–12 at 0.6% strain, a tensile modulus ≈3.2 MPa, and hysteresis of 7.7%. When positioned around the chest, the sensors monitored breathing in real time. Overall, the optimized interplay between ink rheology and conductive network morphology enables the fabrication of strain sensors with high performance and excellent cycling stability (>10 000 compression and 7000 tension cycles). This study establishes a scalable route to solvent‐safe, carbon‐based inks for the sustainable production of flexible and wearable electronics.

## Graphical abstract

An eco‐friendly ink combining carbon nano‐onions, carbon nanotubes, and a SEBS elastomer is formulated in biomass‐derived 2‐MeTHF. Dip‐coated and blade‐coated strain sensors show high conductivity, mechanical flexibility, and stable performance under repeated compression and tension. The ink enables scalable fabrication of sustainable, flexible, and wearable electronic sensors for motion and physiological monitoring.

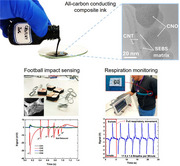

## 1 Introduction

Flexible electronics have attracted considerable interest in a wide range of lightweight and portable applications.^[^ ^1^, ^2^ ^]^ These include wearable healthcare sensors, compact power sources for energy storage, electronic skin, and soft robotics.^[^ ^3^, ^4^, ^5^ ^]^ Piezoresistive and textile sensors detect pressure, strain, or deformation (e.g., stretching or bending) by monitoring subtle changes in ionic^[^ ^6^ ^]^ or electrical resistance linked to modification of the conductive pathways of the material.^[^ ^7^ ^]^ These sensors are suitable for touch‐sensitive devices, human motion detection, and wearable pressure monitoring for human health and physical activity tracking.^[^ ^8^, ^9^ ^]^ In these applications, the capacity to withstand bending, folding, and stretching is paramount. Printing methods offer a scalable and cost‐effective production approach for flexible consumer electronics. Customized inks can be deposited via dip, spray, or blade coating on several surfaces (e.g., polyurethane (PU) foam, fabric, wood, glass, or plastic).^[^ ^10^, ^11^ ^]^ Metal‐based inks (particularly Cu‐based inks) are the standard for the fabrication of conductive components for flexible electronics such as piezoresistive and textile sensors. Although these inks offer high conductivity and low cost, combined with compatibility with large‐scale manufacturing processes,^[^ ^12^ ^]^ they present several key limitations: i) tendency to oxidize, which compromises long‐term reliability;^[^ ^13^ ^]^ ii) limited mechanical compliance to stress, which affects durability;^[^ ^14^ ^]^ and iii) significant toxicity, which hinders their application in wearable motion sensing.^[^ ^15^ ^]^ In general, a main challenge when printing conductive components on flexible or stretchable substrates is the potential mismatch in mechanical properties, which can lead to cracks or fractures and impair conductivity even at low strain levels.^[^ ^14^ ^]^ Another challenge is the incorporation of conductive components into matrix materials or textile fibers, as electrical pathways must maintain structural integrity while progressively deforming under strain and stress.^[^ ^16^, ^17^, ^18^, ^19^ ^]^

To circumvent these issues, composite inks consisting of conductive materials embedded in a soft polymer matrix can produce conformal coatings that adapt and stretch as much as the soft substrate does, preventing the formation of cracks during mechanical deformation.^[^ ^6^, ^20^, ^21^, ^22^ ^]^ Composite inks based on nanocarbons and elastomers represent an ideal alternative to metal‐based inks (due to their high electrical conductivity, lightness, low density, high strength, flexibility, non or limited toxicity, and chemical inertness),^[^ ^23^, ^24^, ^25^, ^26^, ^27^ ^]^ as demonstrated in different types of flexible sensors (e.g., piezoresistive, strain,^[^ ^28^ ^]^ and temperature^[^ ^29^ ^]^), particularly for wearable sensing and human motion monitoring.^[^ ^30^ ^]^ Ensuring a uniform and stable dispersion of conductive nanocarbons at high loadings remains a major obstacle to achieving robust electrical conductivity in strain‐sensing systems.^[^ ^31^ ^]^ One successful strategy involves combining nanocarbons of different dimensionalities (e.g., nanotubes, graphene derivatives, and nano‐onions) to exploit their synergistic effects on mechanical, thermal, and electrical properties.^[^ ^32^, ^33^, ^34^, ^35^, ^36^ ^]^ In particular, carbon nanotubes (CNTs) stand out because of their remarkable electrical conductivity (up to 108 S m^−1^), high thermal conductivity (2000–6000 W m·K^−1^), and excellent mechanical strength.^[^ ^37^, ^38^, ^39^, ^40^, ^41^ ^]^ In composites, their high aspect ratio can simultaneously ease electron transport and reinforce the polymer matrix, producing more durable sensing elements.^[^ ^42^, ^43^ ^]^. 0D nano‐onions (CNOs) with a spherical morphology and high specific surface area (up to 984 m^2^ g^−1^) can further increase conductivity by filling voids between CNT networks.^[^ ^44^, ^45^ ^]^ In addition, they can be produced at low cost and easily dispersed in solvents and polymers, which is ideal for ink production at the industrial scale.^[^ ^46^, ^47^ ^]^ CNOs stand out due to their potential for greener synthesis. They can be obtained by different approaches^[^ ^48^ ^]^ and also derived from renewable sources—including biomass—offering a lower environmental footprint and cost‐effective alternatives, as supported by recent studies.^[^ ^49^ ^]^ Furthermore, CNOs are more chemically reactive than other carbon nanomaterials due to their strained curvature and the nature of their bonding. This heightened reactivity facilitates surface modification, enhancing compatibility with various polymers and expanding their applicability across a broader range of nanocomposite systems.^[^ ^50^ ^]^ Recent advances in polymer/carbon‐based sensors underline the value of such multifiller approaches for high‐performance strain detection.^[^ ^51^, ^52^, ^53^, ^54^, ^55^ ^]^ CNT/polydimethylsiloxane (PDMS) hybrids, for example, have achieved tunable gauge factor (GF) and accurate responses to subtle motions.^[^ ^56^ ^]^ Other designs have realized highly stretchable (>100%) sensors with GF above 35 in the 50–100% strain range,^[^ ^57^ ^]^ whereas some formulations offer extreme sensitivity (over 2000 in certain cases) yet remain robust across repeated deformation cycles.^[^ ^58^, ^59^ ^]^ Additional progress has been reported with stencil‐printable CNT adhesives, which can reach sensitivities above 60 to a strain of 40%^[^ ^60^ ^]^ owing to crack propagation and tunneling mechanisms that amplify resistance changes.^[^ ^61^ ^]^ Within this broader scope, poly(styrene‐ethylene‐butylene‐styrene) (SEBS) has emerged as a promising matrix for flexible sensors owing to its elasticity, durability, and chemical stability.^[^ ^11^ ^]^ SEBS‐based strain sensors incorporating carbon black (CB), graphene, or CNTs have shown reliable performance across diverse loading conditions, with GF ranging from ≈15 to ≈198 and stretchability extending to ≈500%.^[^ ^62^, ^63^, ^64^ ^]^ Still, uniform nanocarbon dispersion and long‐term cycling stability remain key technical challenges. Moreover, most current approaches rely on hazardous petroleum‐derived solvents or expensive nanomaterials, limiting sustainability and scalability.

In this work, we developed a partially biobased hybrid composite ink incorporating CNOs and CNTs within a SEBS elastomer matrix. By merging CNOs with CNTs in a single SEBS matrix, we address common dispersion challenges that often affect all‐carbon composites. The CNOs create additional conductive interfaces without compromising the flexibility of the polymer, whereas the CNTs form bridging networks that remain stable under repeated strains. This synergy enables robust percolation pathways and helps reduce defects or agglomerates. Dip and blade coating methods were used to apply the composite ink onto PU sponges and stretchable textiles to fabricate compressive and tensile strain sensors, respectively. The sensors were characterized in detail in the electrical and mechanical domains, including hysteresis and repeatability metrics. The compressive strain sensors, with a GF of ≈1.1 at –80% strain, showed low electrical hysteresis (11.3%) and excellent durability, maintaining stable readings over 10 000 compression cycles at –50% strain, outperforming comparable sensors reported in the literature. These sensors were further validated in a practical demonstration, where integration into a football enabled effective monitoring of contact, rotation, and rebound events. The tensile strain sensors reached a GF in the range of 10–12 for strains between 0.6% and 2%, with an electrical hysteresis of 7.7% and a repeatability of ≈2.9% over 7000 cycles at 6% strain. When integrated into wearable configurations, the as‐prepared tensile sensors accurately tracked respiratory movements, demonstrating clear potential for real‐time physiological monitoring. Unlike widely studied graphene/CB/SEBS composites, the dual‐dimensional CNO/CNT architecture shortens percolation paths, reduces electrical hysteresis, and enables cycling stabilities (>10 000 compression; 7000 tension cycles) that rank among the best reported for printed textile devices. Our approach integrates specific design and manufacturing strategies that offer notable advantages in cost, performance, and scalability. Overall, these results demonstrate the composite's robustness, sensitivity, and versatility for use in flexible electronics and wearable sensors. The use of green solvents and partially biobased conductive materials offers a more sustainable and adaptable approach, supported by a straightforward device design and fabrication process. Moreover, the broad and versatile strategy of integrating CNTs with bio‐derived conductive nanocarbons described in this study can be effectively extended to bio‐waste from diverse sources, including agricultural, forestry, and marine environments.

By leveraging the distinct properties of different nanocarbons in a SEBS matrix, it becomes possible to optimize percolation pathways, balance mechanical resilience with sensing precision, and scale up fabrication processes for wearable electronics, health monitoring, and other demanding applications.

## 2 Results and Discussion

**Figure 1:**
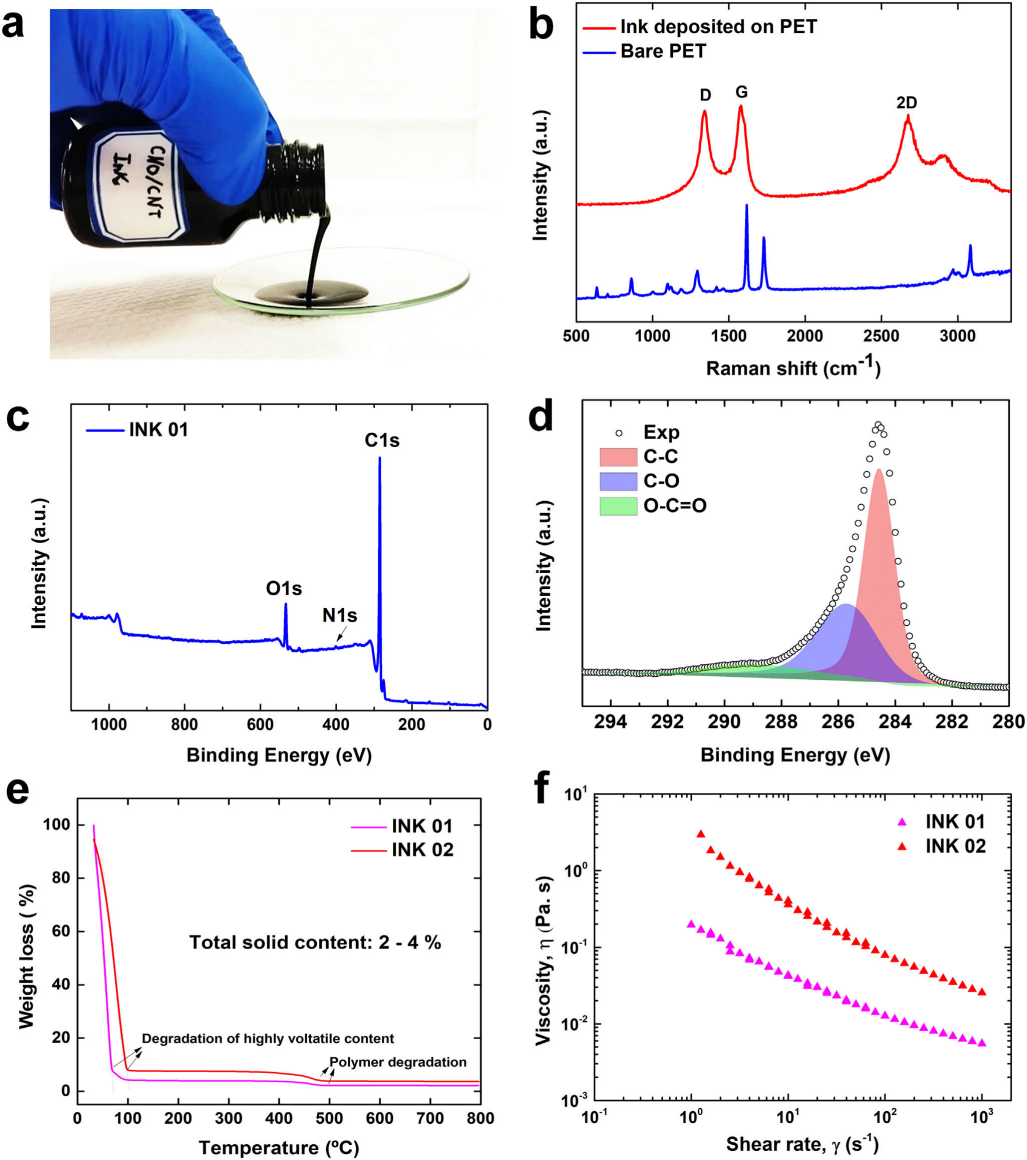
Physicochemical characterization of the nanocomposite ink (INK01): a) photo of the ink; b) Raman spectra; c) XPS survey spectrum; and d) deconvoluted C1s spectrum. e) TGA curves of the two inks in a nitrogen atmosphere (collected at a heating rate of 10 °C min^−1^). f) Shear rate dependence of the viscosity of the inks.

CNOs were synthesized and characterized to determine their physicochemical properties (Figure S1, Supporting Information). As specified in the Experimental Section “Formulation of Inks,” a sequential list of ink formulations was prepared by combining CNOs with different carbon additives in a SEBS matrix (Table S1, Supporting Information). Each ink was deposited on a PET substrate under the same conditions, and the electrical resistance of the conductive pattern was measured. The CNO/CNT composite clearly attained the lowest resistivity. We then performed a detailed characterization of the properties of this ink. **Figure** **1**a shows a photograph of the composite ink, which visually highlights its viscosity. The Raman spectrum in Figure 1b shows main peaks at ≈1340, 1575, and 2672 cm^−1^, corresponding to the D, G, and 2D bands of carbon‐based materials, respectively. The intensity ratio of the D‐band to the G‐band is slightly greater (0.92) than that of the pristine CNO material. The 2D band is more pronounced than that of pristine CNOs, which suggests that the CNOs and CNTs are thoroughly integrated within the SEBS matrix, resulting in a colloidal characteristic of the ink. The chemical composition of the ink was analyzed via XPS. The XPS survey spectrum (Figure 1c) shows the presence of carbon, oxygen, and a negligible amount of nitrogen, possibly originating from the CNTs. The deconvolution of the C1s region (Figure 1d) reveals a prominent sp^2^ peak along with several secondary components associated with carbon and oxides. These findings indicate the absence of any other elemental traces in the ink, confirming its all‐carbon composition. Figures 1e and S2 (Supporting Information) show the TGA curves for the inks in nitrogen and air atmospheres, respectively. In nitrogen, a significant mass loss of 93–96% occurs below 100 °C due to the evolution of the low‐boiling‐point solvent. The much smaller mass loss at ≈425–500 °C is attributed to polymer degradation, which agrees well with the degradation temperature of SEBS in similar nanocomposite systems.^[^ ^65^ ^]^ Above polymer degradation, the mass loss remains almost constant. The residue at 800 °C corresponds to the solid of the carbonaceous materials (CNO/CNT). The final solid content of INK01 is ≈2.2%, in accordance with the ink formulation described in the experimental section. In the case of INK02, where part of the solvent has been removed to concentrate the ink, the solid content is relatively high and reaches 3.7%. The two inks display similar behavior in air, with polymer degradation occurring at lower temperatures (345–385 °C) and the absence of solid residue at 800 °C (Figure S2, Supporting Information). This is a logical consequence of the oxidizing nature of the medium. The rheological behavior of the inks provides key insights into their internal structure and flow characteristics, which are closely related to the solid content. Figure 1f shows that both INK01 and INK02 exhibit non‐Newtonian behavior, as indicated by the shear rate‐dependent viscosity. Both inks display significant shear thinning, with the viscosity decreasing sharply as the shear rate increases. This decrease in viscosity spans nearly two orders of magnitude over the shear rate range explored. However, INK02 consistently has a viscosity approximately one order of magnitude greater than that of INK01. Both inks also exhibit distinct viscoplastic behavior, as evidenced by the increase in the slope of the viscosity curves at lower shear rates.^[^ ^66^ ^]^ This suggests that under low shear, the inks form a more stable and structured microscopic network that resists deformation. In viscoplastic materials, this behavior is often linked to the presence of a yield stress, meaning that a certain threshold of stress is needed before the material starts to flow. The gradual increase in slope at low shear rates likely reflects particle‒particle interactions or the formation of a network within the ink. As the shear rate increases, this network breaks down, leading to the observed shear‐thinning behavior. Thus, the viscoplasticity of both inks is a result of the interplay between their solid‐like structure at rest and their fluid‐like response to higher shear forces, indicating well‐defined microstructural arrangements. This non‐Newtonian and viscoplastic behavior also influence the dynamic moduli, G′ (storage modulus) and G′′ (loss modulus), measured during oscillatory tests. G′ represents the elastic, energy‐storing behavior of the material, whereas G′′ reflects its viscous, energy‐dissipating behavior. In inks with a well‐developed internal structure, such as INK02, a G’ value of ≈3.2 Pa and a loss modulus of ≈2.5 Pa at 0.3 rad s^−1^ were noted, whereas G′ dominates across all frequencies, indicating solid‐like behavior that resists deformation even at low frequencies (Figure S3, Supporting Information). In contrast, INK01 displayed G′ ≈ 0.14 Pa and G″ ≈ 0.16 Pa at 0.3 rad s^−1^, with G′ and G′′ showing similar values at all frequencies, reflecting its more liquid‐like character than INK02. These values reinforce the interpretation of a more robust internal network in INK02 compared with INK01. The weaker structure in INK01 is more prone to flow under low shear conditions, suggesting a greater degree of internal structural breakdown than that in INK02. This could be due to differences in particle interactions, concentration, or network formation and agrees with the different solid contents of the samples. The relatively low G′ in INK01, especially in comparison with that in INK02, suggests a reduced ability to store elastic energy, leading to a more fluid‐like response across the frequency range. Although both inks exhibit shear‐thinning behavior, the dominance of G′′ in INK01 suggests that its internal structure is more easily disrupted, even at low shear rates. As a result, INK01 exhibited more pronounced liquid‐like behavior under stress or deformation.

**Figure 2:**
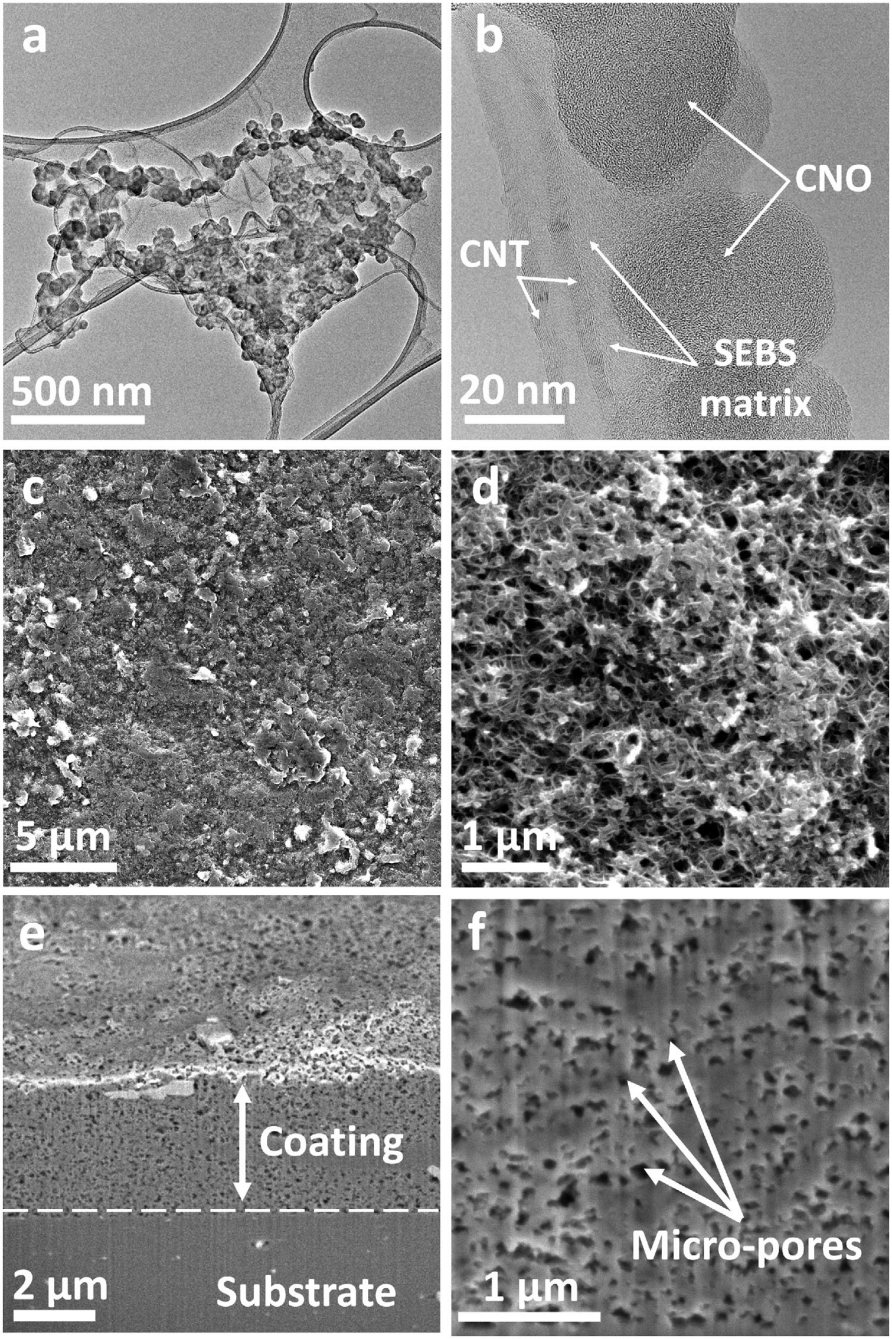
Microstructural analysis of the composite ink and corresponding deposited film. a) Low‐magnification TEM image showing the CNT network partially decorated by quasi‐spherical CNO clusters. b) High‐magnification TEM image revealing individual CNO particles attached to the CNT walls within the SEBS matrix. c,d) Top‐view SEM images of the dried composite film, highlighting a continuous coating with scattered carbon‐rich domains. e) Cross‐sectional SEM image of the film, demonstrating a uniform coating layer atop the substrate. f) Higher‐magnification cross‐section showing the presence of µm‐scale pores (indicated by arrows), which are likely formed during solvent evaporation.

**Figure** **2** shows the morphological features of the CNO/CNT‐SEBS composite ink and its resulting film. In the low‐magnification TEM image (Figure 2a; Figure S4, Supporting Information), a CNT network partially decorated with CNOs can be observed, which appear as clusters of quasi‐spherical particles. At higher magnification (Figure 2b), individual CNOs (with diameters of a few tens of nm) become clearly visible along the CNT sidewalls. The decoration of CNTs with CNOs plays a crucial role for two primary reasons. First, the presence of CNOs reduces the strong van der Waals interactions between individual CNTs, thereby enhancing their dispersion within the polymer matrix. This improved dispersion is evident in the SEBS matrix, which appears as a lighter background in the micrograph, indicating a uniform distribution of nanocarbons throughout the polymer phase. Second, the CNO protrusions on the CNT surface enhance contact between adjacent nanotubes, creating additional conductive pathways without compromising the inherent structure of the CNTs. SEM images (Figure 2c,d) highlight the top surface of a dry film made from the composite ink. The material forms a continuous yet distinctly textured coating, with scattered protrusions corresponding to carbon‐rich regions and occasional pores. This relatively rough landscape implies good filler dispersion, which aids in the creation of a percolated conductive network. The inner structure of the film was further analyzed via cross‐sectional SEM. The dried composite film is ≈4 µm thick and appears in close adhesion to the substrate (Figure 2e). Upon closer inspection, the film reveals µm‐scale pores that likely arise from solvent evaporation during drying (Figure 2f). Such pore formation may enhance flexibility while maintaining conductivity, as the CNO/CNT network remains continuous within the elastomer matrix. Overall, these images confirm that the hybrid ink successfully embeds CNOs and CNTs in SEBS, forming a mechanically robust yet highly conductive coating.

**Figure 3:**
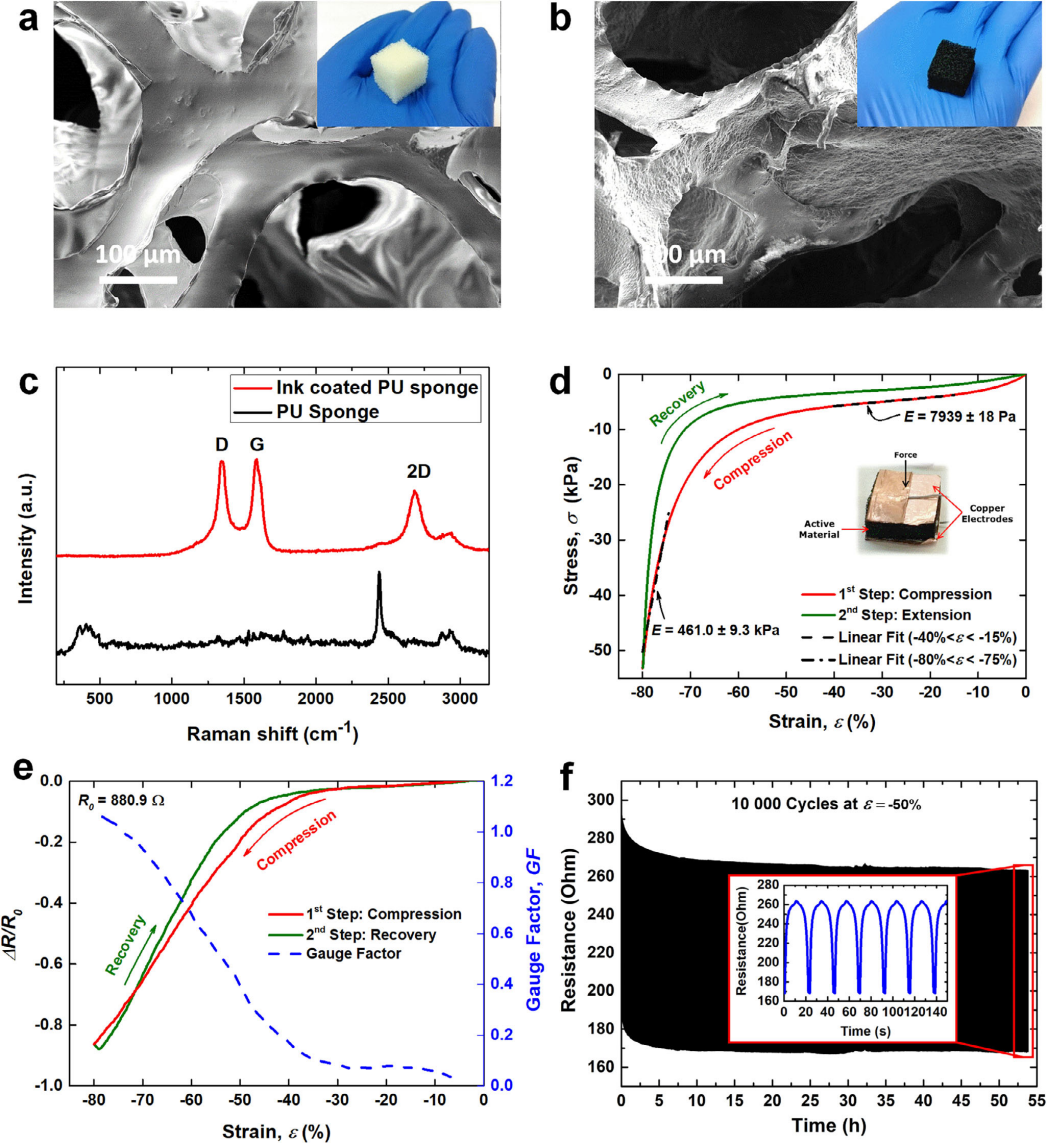
Physicochemical and electrical characterization studies of the CNO/CNT‐SEBS‐coated PU foam: SEM images of the a) original and b) coated PU foam. Photographs are given in the inset. c) Raman spectra. Mechanical and electrical characterization of compressive strain sensors: d) stress versus strain curve, with sensor photo and values of the compressive modulus; e) relative resistance variation and calculated GF during the compression phase, plotted as a function of strain; f) sensor reliability over 10 000 compression–release cycles.

As described in the Experimental Section “Device Fabrication,” PU sponges were dip‐coated with INK01 to fabricate compressive strain sensors. **Figure** **3**a,b shows SEM images and photographs of the sponges before and after ink deposition. Both SEM images show the highly porous network structure of the PU sponge. However, the coated sponge appears to be covered by a thin carbonaceous film, unlike the original sponge, which has a smooth surface. Figure S5a,b (Supporting Information) display high‐resolution SEM images, which confirm that the CNO/CNT‐SEBS composite completely covers the porous PU network. Figure 3c presents the Raman spectrum of the coated PU foam, which aligns well with Figure 1b. This confirms that no compositional changes in the composite occurred after deposition. The compressive strain sensor was tested as described in the Experimental Section “Device Fabrication.” The sponge material exhibits overall mechanical nonlinear behavior with increasing stiffness (Figure 3d), which is typical of foams and rubber for fast load‒unload cycles.^[^ ^67^, ^68^, ^69^ ^]^ In the ranges −40% < *ɛ* < −15% and −80% < *ɛ* < −75%, the stress‒strain response follows a linear behavior, allowing us to perform linear fits in these regions to estimate the compressive modulus of the sensor. The resulting values for the compressive modulus were *E* = 7939 ± 18 Pa and *E* = 461.0 ± 9.3 kPa. The maximum difference in stress between the loading and unloading phases of the cycle is 12.8 kPa (at *ɛ* = −76.6%), which corresponds to *H_M_* = 24.1%. Regarding the electrical response (Figure 3e), the maximum difference in resistance between the loading and unloading parts of the cycle is 89 Ω (at *ɛ* = −55.2%), resulting in *H_R_* = 11.3%. The *GF*, obtained from the loading part of the cycle, is relatively low (≈0.1) for small compression (*ɛ* > −35%), but as the compression is increased toward *ɛ* ≈−80%, the *GF* peaks at ≈1.1 (Figure 2e). The sensor repeatability was tested by performing 10 000 cycles at a peak strain of −50%, with a cycling period of 23.1 s (Figure 3f). The cycling test effectively involves an aging process, which results in a minimum amount of drift in the sensor response over time. The initial resistance drift observed during the first 10 h of cycling is likely due to the microstructural rearrangement and stabilization of the conductive network within the composite, as reported in similar systems.^[^ ^70^, ^71^ ^]^ After the initial 10‐h period, the standard deviation of the resistance at *ɛ* = −50% was only 0.4 Ω, indicating excellent repeatability. This enhanced sensor robustness is attributed to the beneficial role of CNOs in promoting uniform filler dispersion, which contributes to sustained mechanical stability over extended use.

A comparative analysis of sponge‐based piezoresistive strain sensors was performed (**Table** **1**). While the GF of our sensor, ≈1.1 at −80% strain, is in line with the range observed in similar systems, our sensor stands out for its exceptional stability over 10 000 cycles at −50% strain. This endurance and excellent repeatability surpass those of other PU‐based nanocarbon sensors, which typically show higher resistance drift or fatigue over repeated cycles. A recent study by Gong et al. reported the development of hierarchical structures of CB on microporous elastomers, which were used to fabricate compressive strain sensors with a GF and durability exceeding that of our sensors.^[^ ^72^ ^]^ They used high‐temperature melt compounding to produce polymer blends of ethylene‐α‐octene random copolymer (ORC) and a composite of polyoxyethylene and CB. The latter polymer acts as a sacrificial component that can be removed by a long washing process, leaving the CB deposited on the walls of the generated pores. In contrast to our simple, fast, and scalable approach, this strategy is much more complex and difficult to scale up. Additionally, most comparable systems, such as those based on multiwalled CNTs and reduced graphene oxide (MWNT‐rGO), often require more complex or costly material synthesis and processing.

The piezoresistive response of the coated PU sponge arises from pressure‐induced changes in the percolation network of conductive pathways, including the formation and modulation of contact resistance and tunneling junctions between adjacent conductive regions, a mechanism consistent with previous studies on hierarchical CNT composites and microstructured carbon‐based sensors.^[^ ^73^, ^74^ ^]^ Additionally, the effect of the density, pore size, and pore shape of the PU sponge used in our work was not fully taken in consideration with regards to the compressive strain sensor performance – there is room for further simulation and optimization to decrease the sensor hysteresis and increase the GF while preserving the high resistance to fatigue.

**Table 1:** Comparative study of reported piezoresistive strain sensors.

Base material	Conductive material	GF	Endurance	Refs.
PU sponge	Graphene nanosheets		10 000	[75]
PU sponge	Carbon black	3.1 for 50% strain	50 000 at 40% strain	[76]
PU sponge	MWNT‐rGO	−2.3 for 82% strain	5000 at 60% strain	[77]
PU sponge	polyvinyl alcohol (PVA) and CaCl_2_	–	1000 at 60% strain	[78]
Natural sponge	polydopamine rGO and silver nanowires	1.5 for 60% strain	7000 at 60% strain	[79]
PU sponge	MWCNT‐rGO	1.75 for 50% strain	–	[80]
PU sponge	CB/GNPs	–	10	[81]
Melamine sponge	Graphene MWNT	1.99 for 50% strain	12 at 50% strain	[82]
PU	Carbonized wood cellulose sponge	165 (pressure range 0.01–0.5 kPa); 0.29 (3–20 kPa)	6000 at 1 kPa, low hysteresis,	[83]
PU	MWNTs	≈67 (0.3–7 kPa)	>8000 (0.7–3 kPa), hysteresis = 5.2–17.3%, minimum repeatability = 13.1%	[84]
Thermoplastic PU	SWNTs	7 × 10^−4^–0.02 (0.055–255 kPa)	>20 000	[85]
Thermoplastic PU	CB	0.01–1.12 (20–1200 kPa)	10 000 at 30 kPa	[86]
Polydimethylsiloxane/Thermoplastic PU	Ag microflakes	5.54 kPa^−1^ (<10 kPa); 0.123 kPa^−1^ ((10–100 kPa) and 0.0048 kPa^−1^ (100–800 kPa)	>10 000 (20–200 kPa)	[87]
Ethylene‐α‐octene random copolymer (ORC)	CB	30 at 20% strain	13 000 at 20% strain	[72]
PU sponge	CNT/GNPs	2.1 at 20% strain; 1.3 at 50% strain	3000 at 30% strain	[88]
PU sponge	AgNPs/CNTs/CNCs	17.1 at strains lower than 1%	150 cycles at different strains (1–80%)	[89]
PU sponge	AGNPs/SLG	0.47 (<30% strain); 0.66 (30–50%); 2.39 (50–90%)	200 cycles	[90]
PU sponge	Mxene/CNTs	0.58 (0–19% strain); 1.66 (19–60%),	5000 cycles at 60% strain	[91]
PU sponge	CB/CNFs	0.02–0.18 (0‐55% strain)	20 cycles up to 50% strain	[92]
Thermoplastic PU	Branched CNTs	1.5 (up to 60% strain)	200 cycles (0–60% strain)	[93]
PU sponge	rGO/chitosan	0.53–4.62 (0–20% strain)	1000	[94]
PU sponge	CNO‐CNT	1.08 for 80% strain	10 000 at 50% strain	This work

Abbreviations: CB: carbon black; rGO: reduced graphene oxide; G‐NPs: graphene nanoplatelets; SLG: single‐layer graphene; MWNTs: multiwalled carbon nanotubes; SWNTs; single‐walled carbon nanotubes; CNTs: carbon nanotubes (type not specified); CNCs; cellulose nanocrystals; CNFs; cellulose nanofibers.

**Figure 4:**
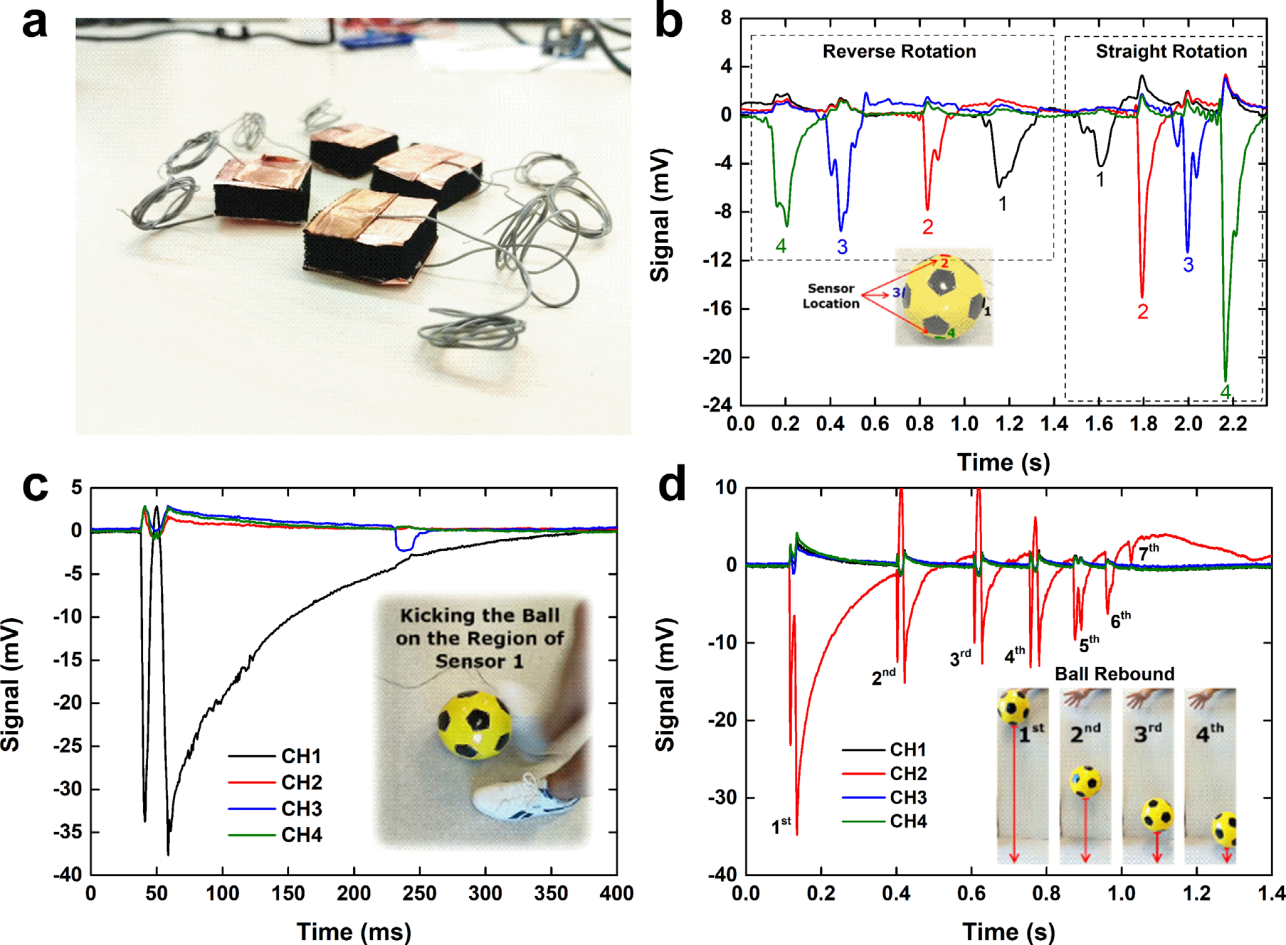
Mechanical and electrical characterization of the sensorized ball. a) Photo of the designed compressive strain sensor devices. b) Sensor response to the rotation of the sensorized ball. The inset shows the sensor location in the ball. c) Kicking of the sensorized ball and sensor response over time. d) Sensor array response over time to ball rebound when it is dropped from a certain height.

Ultimately, the sensor performance depends on both the mechanical properties of the substrate and the composite coating. The outstanding mechanical performance characteristics (low compressive modulus in the range of 1–460 kPa) of our sponge‐based CNO/CNT‐SEBS composite sensor make it ideal for integration into flexible objects or textile substrates for man‒machine interfaces (see Figure S7, Supporting Information for a simple example) and movement monitoring in medical or fitness/sports applications. The stability of our sensor is especially suitable for long‐term and highly repetitive applications. To demonstrate this, we implemented this type of compressive strain sensing system in a football, a scenario in which the sensor is subjected to large and rapid mechanical loads (details in Experimental Section “Device Fabrication” and Figure S6, Supporting Information). **Figure** **4**a displays a photo of the four strain‐sensing devices used for this experiment. Once the four sensors had been mounted and the ball was inflated, the sensor resistance was 10–15 Ω. The ball was placed on the floor and rotated back and forth to identify the temporal sequence of actuation of each sensor and determine the direction of rotation (Figure 4b). The linear speed of the ball was calculated as 0.48 m s^−1^ for the reverse rotation test and 0.84 m s^−1^ for the straight rotation test. A second test was conducted by kicking the ball in the sensor 1 region. While this sensor responded with a greater amplitude, the other three sensors also detected ball deformation (Figure 4c). Notably, the signal from sensor 1 has the opposite polarity relative to those of the other sensors. This suggests that as sensor 1 was compressed, the others were slightly relaxed due to the elastic deformation of the ball. After ≈350 ms, the sensor recovers and returns to its original pre‐strained position. The final test involved dropping the ball from a waist height and making it bounce (Figure 4d). Sensor 2 was positioned at the lowest point of the ball, therefore experiencing the greatest compression and generating the largest signal. As the height of the ball decreases after each rebound, due to energy dissipation, the amplitude of the signal decreases (less force results in less compression), and the time interval between peaks also decreases because of the shorter travel distance. A total of seven rebounds were identified. For both Figure 4c,d, the peak of the most intense impact seems to echo, showing two similar peaks immediately after one another. This is due to the movement of the bladder inside the ball, which is not stuck to the surface of the ball and therefore makes an independent movement, compressing and relaxing with a small delay during impact.

**Figure 5:**
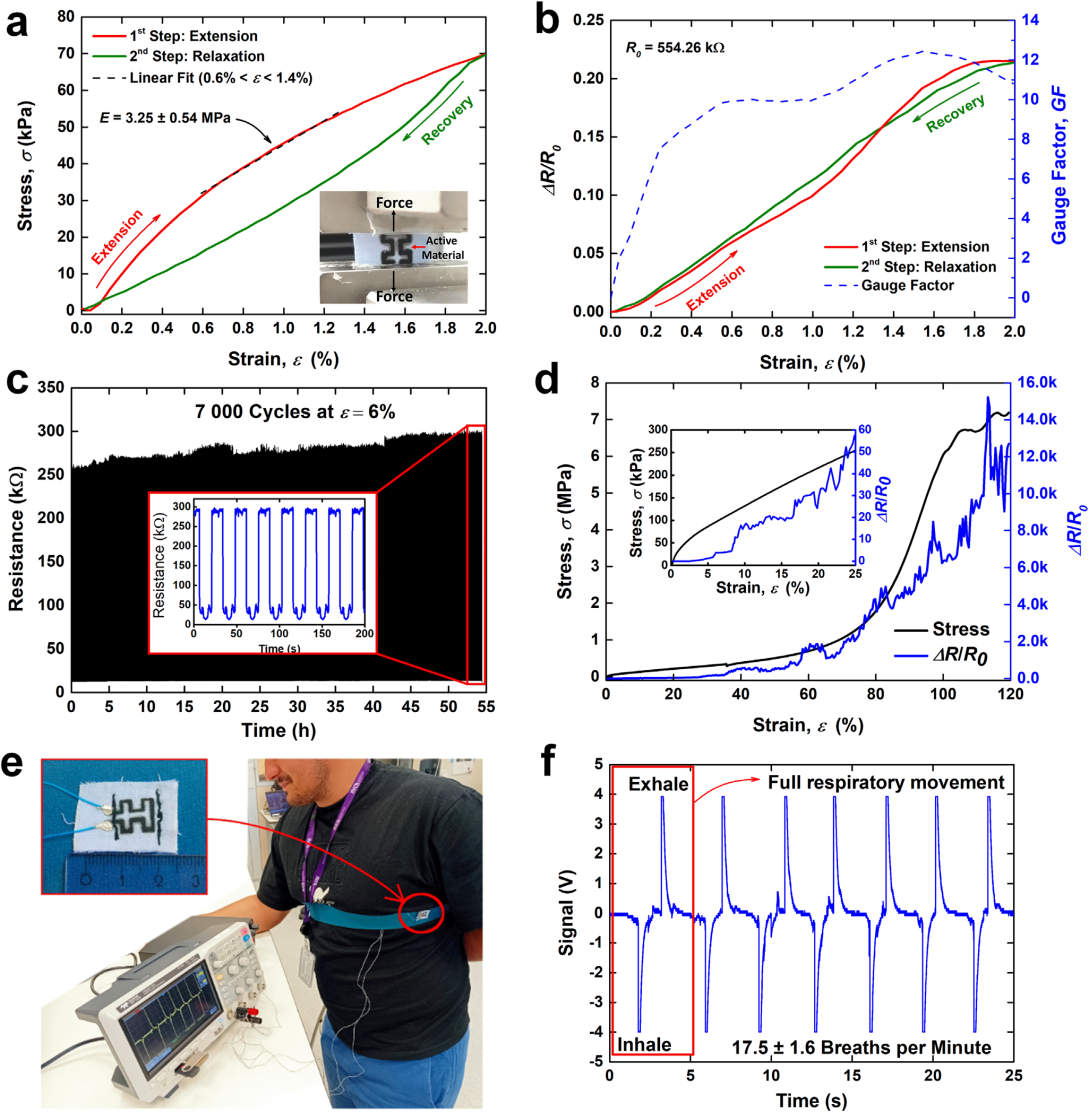
Electromechanical characterization of the developed CNO/CNT‐based tensile strain sensor. a) Stress‒strain curve. The inset shows a picture of a typical sensor under test (mounted in the universal testing machine), and the graphic also contains the calculated modulus value. b) Relative resistance variation and calculated GF during the extension phase, plotted as a function of strain. c) Sensor repeatability test over 7000 cycles at 6% strain. d) Destructive test performed on a tensile sensor until ɛ = 120%. e) Mounting of the sensor on an elastic band for breath movement recording (the active region of the sensor is 11 mm long (between stitches)). f) Sensor signal during breathing (the signal variation is inversely proportional to the resistance variation).

Finally, a CNO/CNT‐based tensile strain sensor was fabricated on elastic fabric (Figure S7a–c, Supporting Information) and tested, as described in Experimental Section “Device Fabrication.” The sensor displayed typical nonlinear mechanical behavior with some hysteresis (**Figure** **5**a), which is characteristic of woven fabrics.^[^ ^95^, ^96^ ^]^ A linear fit was performed on the stretching portion of the curve (within the range of 0.6% < *ɛ* < 1.4%), which yielded a tensile modulus of *E* = 3.25 ± 0.54 MPa. The maximum stress difference between the extension and recovery phases was 16.7 kPa (ɛ = 0.94%), corresponding to *H_M_* = 23.9%. Regarding the electrical response (Figure 5b), the maximum resistance difference between the loading and unloading phases was 9.1 kΩ (at *ɛ* ≈ 1%), resulting in an *H_R_* = 7.7%. Notably, both the mechanical and electrical hysteresis peaks occurred at approximately the same strain (*ɛ* ≈ 1%). While the *GF* was low for ɛ < 0.4%, it increased to 10–12 in the range of 0.6% < *ɛ* < 2%. The sensor's repeatability was evaluated through the completion of 7000 cycles at a peak strain of 6%, with a cycle duration of 27.6 s (Figure 5c). During the 55‐h test, the sensor resistance increased from ≈250 to 280 kΩ. Throughout the course of the experiment, the standard deviation of the resistance at *ɛ* = 6% was found to be 8.5 kΩ, resulting in a repeatability of *δR* ≈ 2.9%. Although the sensor exhibits a drift, which can be related to the interaction between the ink and the substrate, this clearly demonstrates an improvement in repeatability compared with a similar sensor made with graphene paste, where *δR* was ≈5.2%.^[^ ^30^ ^]^ For further optimization of the sensor response and elimination of the time‐dependent drift, a more detailed study of the effect of the substrate and coating thickness have to be performed. To evaluate the sensor reliability under high strain, continuous stretching was performed while monitoring the resistance (Figure 5d). For *ɛ* > 8%, significant and unpredictable fluctuations in resistance were noted, which are likely due to the formation of cracks in the active material. For this type of textile or active material, the safe operating range is clearly below *ɛ* < 10%. At *ɛ* ≈ 100%, the sensor begins to slip from the mounting point on the testing machine, resulting in the applied force reaching a plateau. The test was terminated at this point, and the sensor did not recover after such extreme strain. To demonstrate the potential for wearable and sports applications, where low‐level strain sensitivity and long‐term stability are required, a tensile sensor was attached to an elastic band and placed around the chest of a test subject to monitor breathing movements (Figure 5e). The inhalation and exhalation phases were clearly distinguishable in the measured AC voltages (Figure 5f). By analyzing the time interval between inhalation peaks, a respiratory rate of 17.5 ± 1.6 breaths per minute was determined. These results demonstrate the potential of the tensile strain sensor for monitoring various body movements or vital parameters and its suitability for integration into wearable sensor systems.

When our CNO/CNT‐SEBS textile sensor is compared with similar devices from the literature (**Table** **2**), several key advantages stand out. With a GF of 10–12 at low strains (0.6–2%) and stability across 7000 cycles at 6% strain, our sensor combines high sensitivity with excellent repeatability (2.9% drift), comparable or even superior to many reported textile and elastomer‐based sensors. For example, CNT‐ and GO‐based fabrics show either lower GF or reduced durability under repeated strains, which can limit their wearable applications. Table 2 shows high GF values for the most relevant sensors.

**Table 2:** Comparative study of reported flexible stretchable sensors.

Filler type	Composite/device fabrication	Sustainability aspects	GF	Endurance	Refs.
MWNTs	Elastic fabric	–	–	15 for 60% strain	[97]
Conductive carbon	90%polyester/10% spandex fabric	–	–	250 for 30% strain	[98]
Carbon grease	i) elastomeric component part mixing and pouring on a petri dish; ii) conductive ink homogenization in planetary mixer; iii) embedded 3D printing of conductive ink with a syringe	–	3.8 for 16.7% strain	1000 for 1% strain	[99]
MWNTs	Ecoflex	–	1.75 for 40% strain	2000 for 300% strain	[100]
Carbon black	i) solution mixing of conductive elastomer; ii) Casting the bottom silicone elastomer; iii) conductive elastomer; iv) laser‐ablating the electrode layer pattern; v) casting the top elastomer layer	–	3.37 for 50% strain	10 100 for 200% strain	[101]
GO	i) immersing textile in GO solutions and drying (three times); ii) annealing at 200 °C; iii) mixing with PDMS; iv) curing at 60 °C, 12 h	High temperature, energy consumption	−26 for 6% strain	500 for 5% strain	[102]
rGO/SWNTs	i) dipping textile in GO solution and drying at 80 °C (Five times); ii) chemical reduction overnight, 80 °C; iii) dipping into SWMTs and drying at 80 °C (five times)	Hazardous chemicals, hydrazine, energy and time consumption	6.1 for 3.3–5.5% strain	10 000 for 11.6% strain	[103]
rGO	Ultrasound‐assisted GO dispersion in water; iii) vacuum filtration through textile; iii) hot pressing at 180 °C	Hazardous chemicals for GO synthesis, high temperature for GO reduction	–	400	[104]
CNT	i) Polymer dissolving; ii) mixing with CNT; iii) spraying layer‐by‐layer	Hazardous chlorinated solvent, chloroform	2.5 for 0.45–0.6% strain	400 for 0.65% strain	[105]
rGO	i) Ultrasound‐assisted GO dispersion in water; ii) textile dipping and drying (six times); iii) chemical reduction and drying	Hazardous chemicals, sodium borohydride	18.5 for 10% strain	120 for 3% strain	[106]
VGCNFs	i) High shear mixing in a planetary mixer; ii) casting and drying; iii) melt pressing at 190 °C	Hazardous petroleum‐based solvent, toluene; high temperature.	≈4 at 10 and 20% strain	50 cycles at 50% strain	[107]
CB	i) Melt compoiunding; ii) mixing by melt compounding; iii) compression molding; iv) washing (24 h) and drying	Energy and time consuming	Up to 1000 at 300% strain	2000 cycles up to 50% strain	[72]
CNT/GNPs	i) Dispersion carbon nanomaterials; ii) electrostatic layer‐by‐layer assembling (6 h each step)	Hazardous chemicals, sodiumdodecylbenzenesulfonate (SDBS), N,N‐di (2‐aminoethyl)‐perylene‐3,4,9,10‐tetracarboxylicdiimide (AE‐PTDI)	>7000 at 25% strain	3000 cycles at 30% strain	[88]
CNTs/CNOs	Ink prepared by a single mixing step involving CNT, CNO, and elastomer.	Biobased solvent; partially biobased conductive filler	10‐12 at 0.6‐2% strain	≈7000 at 6% strain	This work

Nonlinear behavior is frequently observed in these devices, making the *GF* closely dependent on the applied strain. This complicates direct comparisons among devices based on different composites (with many variables such as the composition, morphology, and content of fillers and polymeric matrix).^[^ ^108^ ^]^ Our sensor shows the highest endurance among all the reported systems in terms of the number of stretching cycles, signal stability, and electrical hysteresis (often not reported in the literature). Because they are uniformly dispersed in the SEBS matrix, the combination of interconnected 1D CNT pathways and 0D CNO bridging points establishes a resilient percolation network throughout the elastomer in both sensor types. Under compressive loading, the skeleton of the PU sensor contracts and pushes filler‐rich regions closer together, increasing the number of CNO‐CNT contact points and thus increasing the electrical conductivity. Under moderate tensile loading, the textile‐based sensor generally retains conductive pathways; however, larger strains can eventually disrupt those filler connections, increasing resistance. This direct coupling between the filler arrangement and mechanical deformation explains each sensor's characteristic GF, hysteresis, and repeatability. The elastomer's elasticity accommodates substantial deformation, whereas the well‐dispersed nanocarbon fillers convert subtle microstructural changes into stable, measurable conductivity variations. In addition to these parameters, other key features, such as sustainability, versatility, and washability, need to be considered in recent studies.^[^ ^108^, ^109^, ^110^, ^111^, ^112^ ^]^ In this regard, our sensors unlock significant advancements over existing systems owing to the use of green solvents and partially biobased conductive materials, with a simple device configuration and fabrication procedure. Notably, our sensors were tested for stability after “real” washing conditions (i.e., comparable to conventional, home‐washing cycles), resulting in a minimal resistance variation below 5% after ten cycles (Figure S7d, Supporting Information). Owing to the demonstrated robustness and resistance to washing, we believe that the CNO/CNT‐SEBS sensing system could be implemented in real‐world applications and integrated into commercial textiles.

## 3 Conclusion

In this study, we developed a nanocomposite ink by combining CNTs with CNOs, synthesized via a simple flame‐based method. These fillers were embedded in a SEBS matrix, yielding a cost‐effective, sustainable, and readily scalable solution with strong potential for industrial production. Formulated in biomass‐derived 2‐MeTHF, the ink can be readily deposited via dip, blade, or spray coating. Its non‐Newtonian behavior provides tunable viscosity while preserving high conductivity. We optimized the formulation for strong adhesion to polyurethane and textile substrates, allowing the facile fabrication of proof‐of‐concept devices. The inks demonstrated high versatility, allowing the preparation of sensing systems to monitor parameters in very different scenarios such as large and rapid mechanical deformation for impact detection as well as low‐magnitude, cyclic stretching for health monitoring. Polyurethane sponges were transformed into compressive strain sensors via a facile dip‐coating process. Under compressive strain up to −80%, these sensors maintained stable resistivity due to intimate coating–substrate interactions and demonstrated consistency over 10 000 cycles. When integrated into a football, they detected contact, rotation, and rebound, allowing for effective, real‐time motion monitoring. Meanwhile, stretchable textiles were converted into tensile strain sensors by embedding customized conductive patterns via blade coating. The tensile sensor retained a readout repeatability of 2.9% across 7000 cycles at 6% strain, and accurately tracked breathing movements when positioned on an elastic band around the chest area. By leveraging the distinct properties of different nanocarbons in the SEBS matrix, we optimized percolation pathways, balanced mechanical resilience with sensing precision, and enabled scalable fabrication for wearable health and motion monitoring. These CNT/CNO–SEBS devices combine high gauge factors, long‐term stability, and environmental resistance, confirming robustness and reliability for daily‐use applications. The CNTs create primary conductive channels due to their high aspect ratio, while CNOs occupy interstitial gaps, enhancing percolation and resulting in a uniformly distributed network. This dual‐filler synergy facilitates efficient electron transport even under mechanical stress. Compared with other flexible and stretchable materials—many of which rely on expensive or hazardous components—our composite inks offer a more versatile and sustainable alternative. Their compatibility with standard printing methods and customizable rheology makes them suitable for 3D printing and other large‐scale fabrication techniques. This study also reveals new possibilities for revalorizing bio‐waste into flexible conductors. While focused on a specific bio‐derived route for CNO synthesis, the approach—combining bio‐based conductive nanostructures with CNTs—can be extended to a wide range of agricultural, forestry, and marine residues, broadening the potential for high‐value reuse of abundant waste materials.

## 4 Experimental Section

### Material Details

Clarified butter (from the local market), MWCNTs (Nanocy, NC7000—9.5 nm average diameter, 1.5 µm average length, 90% carbon purity, 250–300 m^2^ g^−1^ surface area, 10^−4^ ohm cm resistivity), and 2‐methyltetrahydrofuran (673277) from Sigma Aldrich were used. Styrene‐ethylene‐butylene‐styrene thermoplastic elastomer (SEBS) was provided by Dinasol (Madrid, Spain). The SEBS grade employed in this work contains 30 wt.% styrene, a weight‐average molecular weight of Mw = 85 000 g mol^−1^ (determined by GPC) and a polydispersity index of Mw/Mn = 1.45. Commercial PU foam and commercial stretchable fabric (composed of 82% cellulose and 18% polyamide) were used as substrates for INK01 and INK02, respectively.

### Preparation of CNOs

Pristine CNOs were prepared via an approach described in the previous publications.^[^ ^113^, ^114^ ^]^ CNOs were synthesized via an economical and straightforward flame‐based method. In brief, a cotton wick, ≈4–5 mm in diameter, was used to sustain a flame fueled by clarified butter, which served as the carbon precursor. Through capillary action, the precursor was drawn into the wick and combusted in a controlled, wind‐free environment. To gather the resulting soot, a clean copper foil was positioned 2 cm above the flame, ensuring efficient deposition of the carbon material. The obtained black carbon CNO nanoparticles were collected and utilized for developing nanocomposite inks without the need for post‐processing treatments. 100 mL of pristine CNO powder was dispersed in 20 mL of ethanol for characterization.

### Formulation of Inks

Inks with different formulations (i.e., combinations of CNO with various carbonaceous materials) were prepared. The electrical resistance of blade‐coated strips (10 µm thick, on PET) from these inks was compared to identify the highest conductivity. The full information is included in Table S1 (Supporting Information). The selected formulation corresponds to the sample combining CNO and CNTs, which was prepared according to the following protocol: 370 mg of CNO and 276 mg of CNTs were added to 10 mL of a solution of SEBS in 2 m‐THF (40 mg. mL^−1^), resulting in a highly viscous paste. A further 10 mL of solvent was added to this paste, and the sample was ultrasonicated for 15 min at 0.5 s pulses. Finally, a further 10 mL of solvent was added, and the resulting mixture was stirred magnetically for a period of 72 h, producing a homogeneous ink (named “INK01”). The use of a low‐boiling solvent, as in this case, allows the content of carbonaceous species to be increased by simple solvent evaporation. These changes in solid content have a significant effect on the rheological properties of the ink and consequently on the ability to form coatings via various experimental approaches. Therefore, a second, more concentrated ink (named “INK02”) was prepared. The coating details for both inks are described as follows:

### Formulation of Inks—Dip Coating of PU Sponge

Sponge cubes were subjected to a dip‐coating process by immersion in INK01 for 60 s, followed by withdrawal at a controlled rate of 50 mm min^−1^ and drying at 40 °C for 30 min. The coating cycle was repeated twice to achieve uniform surface coverage prior to subsequent evaluation of coating morphology and electrical performance.

### Formulation of Inks—Blade Coating of Textile

INK02 was deposited onto the textile substrate through a custom‐designed mask using a 150 µm blade gap at a coating speed of 20 mm·s^−1^, followed by drying at 60 °C for 20 min. The mask defined a closed conductive pattern in the shape of an “H” (13 × 9 mm), with a total track length of 50 mm and a width of 0.5 mm. Nickel paste was applied at both ends of the sensor to establish electrical connections with the wires.

### Materials Characterization—Raman Spectroscopy

50 µL of the CNO dispersion in ethanol was drop‐cast onto glass substrates (1 × 1 cm^2^) and dried at 60 °C for 10 min. 100 µL of CNO/CNT ink was drop‐cast on the PET substrate and allowed to dry at 40 °C for 15 min. Pristine PU foam and ink‐coated foam were also used for the analysis. All the measurements were performed with an ALPHA300R confocal Raman microscope (WITec) using 532 nm laser (2 mW power) light for excitation at room temperature.

### Materials Characterization—Scanning Electron Microscopy (SEM)

50 µL of the CNO dispersion in ethanol was drop‐cast on a cleaned Si substrate (1 × 1 cm^2^) and dried at 60 °C for 10 min. INK 02 was deposited on PET substrate using blade coating technique. For the cross‐section analysis, the sample were prepared by a dual‐beam FEI Helios 450S focused ion beam (FIB). A 30 kV ion beam was used for the bulk milling and 3 kV and 100 pA current were employed for imaging by the Secondary electron detector SEM. Images were acquired via an FEI Quanta 650 FEG with a cold field emission electron source using acceleration voltages between 5 and 10 kV at a 10 mm working distance under high vacuum.

### Materials Characterization—Transmission Electron Microscopy (TEM)

A 20‐µL aliquot of the diluted (1:20) CNO dispersion in ethanol was drop‐cast on Cu grids (200 mesh, covered with lacey carbon) and allowed to dry at 80 °C for 30 min. A 20‐µL aliquot of the diluted CNO/CNT (1:50) ink in 2 Me‐THF was drop‐cast on Cu grids (200 mesh, covered with lacey carbon) and allowed to dry at 40 °C for 30 min. The structure of the CNOs and composite ink was investigated via a double‐corrected FEI Titan G3 Cubed Themis instrument operated at 200 kV.

### Materials Characterization—X‐Ray Photoelectron Spectroscopy (XPS)

The same samples used for Raman analysis were used for the XPS measurements. XPS spectra were acquired with an ESCALAB 250 XI (Thermo Fisher Scientific, Source: Al K_α_ 1486.6 eV, 650 µm spot size, Pass energy: 40 eV with hemispherical analyzer) system with an analysis chamber maintained in ultrahigh vacuum (UHV ≈5 × 10^−10^ mbar) conditions.

### Materials Characterization—Thermogravimetric Analysis (TGA)

TGA was used to determine the solid content in the ink. 100 µL of the ink was added to the TGA pan, and the thermograms were recorded with a TA 50 instrument in both nitrogen and air atmospheres. Owing to the low boiling point of the solvent used in the study, an initial purging step was not applied to avoid solvent loss, leading to misinterpretation of the solid content of the ink. The samples were heated at a heating rate of 10 °C min^−1^. The data were analyzed, and the mass loss and degradation temperature were calculated via TA instrument software.

### Materials Characterization—Rheology

The rheological properties of the inks were characterized via a rotational shear test on an HR20 Discovery rheometer (TA Instruments). The device was equipped with a cone‒plate geometry, featuring a 40 mm diameter cone with a 2° angle, and the measurements were performed under both steady and dynamic shear conditions at a controlled temperature of 25 °C. For steady‐state viscosity measurements, constant shear rate mode was applied, covering a range from 1 to 1000 s^−1^. The dynamic rheological tests were conducted over a broad spectrum of angular frequencies, ranging from 0.03 to 100 rad·s^−1^, with a fixed stress of 10 Pa. Each measurement was performed in duplicate to ensure data accuracy and reproducibility.

### Device Fabrication—Compressive Strain Sensor Fabrication and Testing

Cube‐shaped PU sponges (density: 30.031 ± 1.321 kg·m^−3^; active area: 10 mm width × 12 mm depth × 10 mm thickness) were fabricated and dip‐coated in INK01 by immersing for 60 s, withdrawing at 50 mm·min^−1^, and drying at 40 °C for 30 min; the cycle was repeated twice to ensure uniform coverage before morphological and electrical evaluation. Copper foil electrodes were placed on opposite sides of the cube‐shaped sponge sensor across its thickness. Nickel conductive ink (CW2000, Chemtronics) was used to guarantee good electrical contact between the copper electrodes and the porous active material. Compressive forces were applied across the thickness of the sensor. A comprehensive analysis of the sensor performance was conducted to evaluate its mechanical and electrical behavior under compression/release cycles. This assessment enabled the calculation of key metrics, including the compressive modulus (*E*), the gauge factor (*GF*), the mechanical hysteresis (*H_M_*), and the electrical hysteresis (*H_R_*). The mechanical characterization (stress vs strain studies) of both the compressive and tensile strain sensors was performed on a universal testing machine (AGX‐V, Shimadzu Corporation). The compressive strain sensor was tested in the range of −80% < *ɛ* < 0% by applying increasing compression and then returning it to the initial configuration (the linear speed of the test was 0.1 mm s^−1^). In line with standard practice, negative stress and negative strain were defined for compression (and, conversely, positive stress and positive strain for tension). The resistance measurements were performed with a digital multimeter (Agilent 43410A). The sensors were subjected to 10 000 loading cycles, allowing the repeatability of the sensor response (*δ_R_*) to be assessed. For further details on the calculation of these metrics, please refer to previous work from the group.^[^ ^30^ ^]^ The long‐term cycling test (uniaxial stress) was performed using a Thorlabs stage equipped with a linear actuator (Z825B) and the respective controller (KDC101) at a linear speed of 12 mm min^−1^. For application demonstration, the sensor was connected in series with a DC power supply and a fixed resistor (47 kΩ), forming a voltage divider. The voltage across the fixed resistor was measured via an oscilloscope (T3DSO1204, Teledyne LeCroy) in AC coupling mode.

For football sensorization, four compressive strain sensors were installed in a KIPSTA football (67.5 cm circumference) between the ball bladder and the outer layer, resulting in a precompression of the sensors for a higher GF. The sensors (10 mm width × 12 mm depth × 10 mm thickness) were made of coated PU foam sandwiched between two copper foil electrodes. For the resistance reading, the sensors were connected in series with a DC power supply and individual load resistors (5.6 Ω), which were driven with a current of 75 mA each. The AC voltage (using AC coupling) at each resistor of the voltage divider was monitored simultaneously via an oscilloscope (T3DSO1204, Teledyne LeCroy). The signals for each sensor (*V_n_*) were inversely proportional to the resistance variation of the sensor (*R_sensor_*):

(1) $$V_{n}=\frac{5.6}{R_{sensor}+5.6}V$$

In a post‐processing step, the sensor signals were low‐pass filtered at 1 kHz to remove noise.

### Device Fabrication—Tensile Strain Sensor Fabrication and Testing

INK02 was then blade‐coated onto commercial fabric using a customized mask (150 µm gap, 20 mm·s^−1^) to form a closed conductive pattern (design dimensions: 13 × 9 mm, total track length 50 mm, track width 0.5 mm), followed by drying at 60 °C for 20 min. Nickel paste was applied at both ends to connect the sensor to electrical wires. The sensors were fabricated on commercial fabric via blade coating with the same pattern. Nickel paste was applied to connect the ends of the graphene sensor to the electrical wires. The active area measured 25 mm (width) × 11 mm (length) × 360 µm (thickness). Uniaxial stress was applied along the length of the sensor. The sensor was evaluated under different strain conditions to assess both its mechanical and electrical behavior, similar to what was previously described for a compressive strain sensor. The sensor was tested within the range of 0% < ɛ < 2% by gradually applying tension and then returning it to its initial state, with a linear test speed of 0.1 mm s^−1^, via a universal testing machine. The sensors were subsequently subjected to 7000 cycles of cyclic stretching (with a linear speed of 1.4 mm min^−1^) to assess the repeatability of the sensor response (*δR*).^[^ ^16^, ^17^, ^18^, ^19^ ^]^ The resistance to washing was evaluated by immersing the fabric in a polypropylene container with a 1 vol.% solution of a commercial detergent in water and 15 titanium balls with a diameter of 6 mm. The sample was placed in a Thinky ARE‐250 planetary centrifugal mixer (PCM) (Thinky Corporation, Tokyo, Japan), and the following program was applied: 15 min at 400 rpm, followed by 5 min at 800 rpm at 40 °C. The textile was then washed with water and dried at 60 °C under vacuum for 20 min before the resistance was recorded.

## Conflict of Interest

The authors declare no conflict of interest.

## Supporting information



Supporting Information

## Data Availability

The data that support the findings of this study are available from the corresponding author upon reasonable request.
