# Supplementary material for: Electrically Conductive Nanocarbon/Elastomer Composite Inks for Flexible and Wearable Strain Sensing
Source: Small. 2025 Nov 14;21(50):e06844. doi: 10.1002/smll.202506844 (PMC12710138; doi:10.1002/smll.202506844)
Supplement: Supplementary file 1 — Supporting Information [file SMLL-21-e06844-s001.pdf]

NANO · MICRO
small

Supporting Information

for *Small*, DOI 10.1002/smll.202506844

Electrically Conductive Nanocarbon/Elastomer Composite Inks for Flexible and Wearable Strain Sensing

Siva Sankar Nemala, Bruno Bernardino, Rui M. R. Pinto, Vicente Lopes, Pedro Alpuim, Ihsan Çaha, Edoardo Sotgiu, Marián A. Gómez-Fatou, Juan Francisco Vega, Horacio Javier Salavagione and Andrea Capasso**

Electrically conductive nanocarbon/elastomer composite inks for flexible and wearable strain sensing

Siva Sankar Nemala[1], Bruno Bernardino[1], Rui M. R. Pinto[1], Vicente Lopes[1], Pedro Alpuim[1,2], Ihsan Çaha[1], Edoardo Sotgiu[1], Marián A. Gómez-Fatou[3], Juan Francisco Vega[4], Horacio Javier Salavagione[3], Andrea Capasso[1*]*

[1]International Iberian Nanotechnology Laboratory, 4715-330 Braga, Portugal.

[2]Centro de Física das Universidades do Minho e Porto, Universidade do Minho, Braga 4710-057, Portugal

[3]Instituto de Ciencia y Tecnología de Polímeros (ICTP), CSIC. Departamento de Física de Polímeros, Elastómeros y Aplicaciones Energéticas, c/ Juan de la Cierva 3, 28006, Madrid, Spain

[4]Instituto de Estructura de la Materia (IEM), CSIC. Departamento de Física Macromolecular. BIOPHYM. Serrano 113bis, 28006, Madrid, Spain

*Email addresses: andrea.capasso@inl.int (Andrea Capasso), horacio.salavagione@csic.es (Horacio Javier Salavagione)

Supplementary Information

Table S1. List of ink formulation tested in this study. For the resistance measurements the probes were put 1 cm apart.

Carbon materials	Ink formulation	CNO: Additive ratio	Carbon material loading in films	CNO loading in the film	Films resistance
CNO	49 mg CNO; 400 mg SEBS; 5mL 2mTHF	1:0	11 %	11 %	> 1 MΩ
CNO + CB	39 mg CNO; 10 mg CB; 400 mg SEBS; 5mL 2mTHF	4:1	11 %	8.7 %	> 1 MΩ
CNO + CB	30 mg CNO; 20.5 mg CB; 400 mg SEBS; 5mL 2mTHF	3:2	11%	6.7 %	> 1 MΩ
CNO + HCG	39 mg CNO; 10.5 mg HCG; 400 mg SEBS; 5mL 2mTHF	4:1	11%	8.7 %	> 1 MΩ
CNO + HCG	30.5 mg CNO; 21 mg HCG; 400 mg SEBS; 5mL 2mTHF	3:2	11%	6.7 %	> 1 MΩ
CNO + HCG	47 mg CNO; 32 mg HCG; 400 mg SEBS; 5mL 2mTHF	3:2	16.5 %	9.9 %	> 1 MΩ
CNO + C150	142 mg CNO; 95 mg CNT; 200 mg SEBS; 5mL 2mTHF	3:2	45 %	19.4 %	13 KΩ
CNO + CNF (Ganf antolin)	142 mg CNO; 95 mg CNF; 200 mg SEBS; 5mL 2mTHF	3:2	45 %	19.4 %	130 KΩ
CNO + C65 (graphite+CB)	122 mg CNO; 79 mg C65; 200 mg SEBS; 5mL 2mTHF	3:2	50 %	30.4 %	~300-500 Ω
CNO + KS44 (Graphite)	123 mg CNO; 81 mg KS44; 200 mg SEBS; 5mL 2mTHF	3:2	50.5 %	30.4 %	>2 KΩ
CNO + C65	170 mg CNO; 114 mg C65; 200 mg SEBS; 5mL 2mTHF	3:2	58.7 %	35.1 %	200 Ω
**CNO + NC7000**	**182 mg CNO; 136 mg NC7000; 200 mg SEBS; 10 mL 2mTHF**	**3:2**	**61.4 %**	**35.1 %**	**~1-10 Ω**

CNO: carbon nanoonions; ***CB***: Carbon black (Cabot); ***HCG***: highly conductive graphite: ***C150***: MWNT-Baytubes C150 P (Bayern); ***CNF***: Carbon nanofibers, *GANF* (Grupo Antolin); ***C65***: graphite + CB (Timcal). ***KS44***: graphite (Timcal); ***NC7000***: MWNT (Nanocyl).

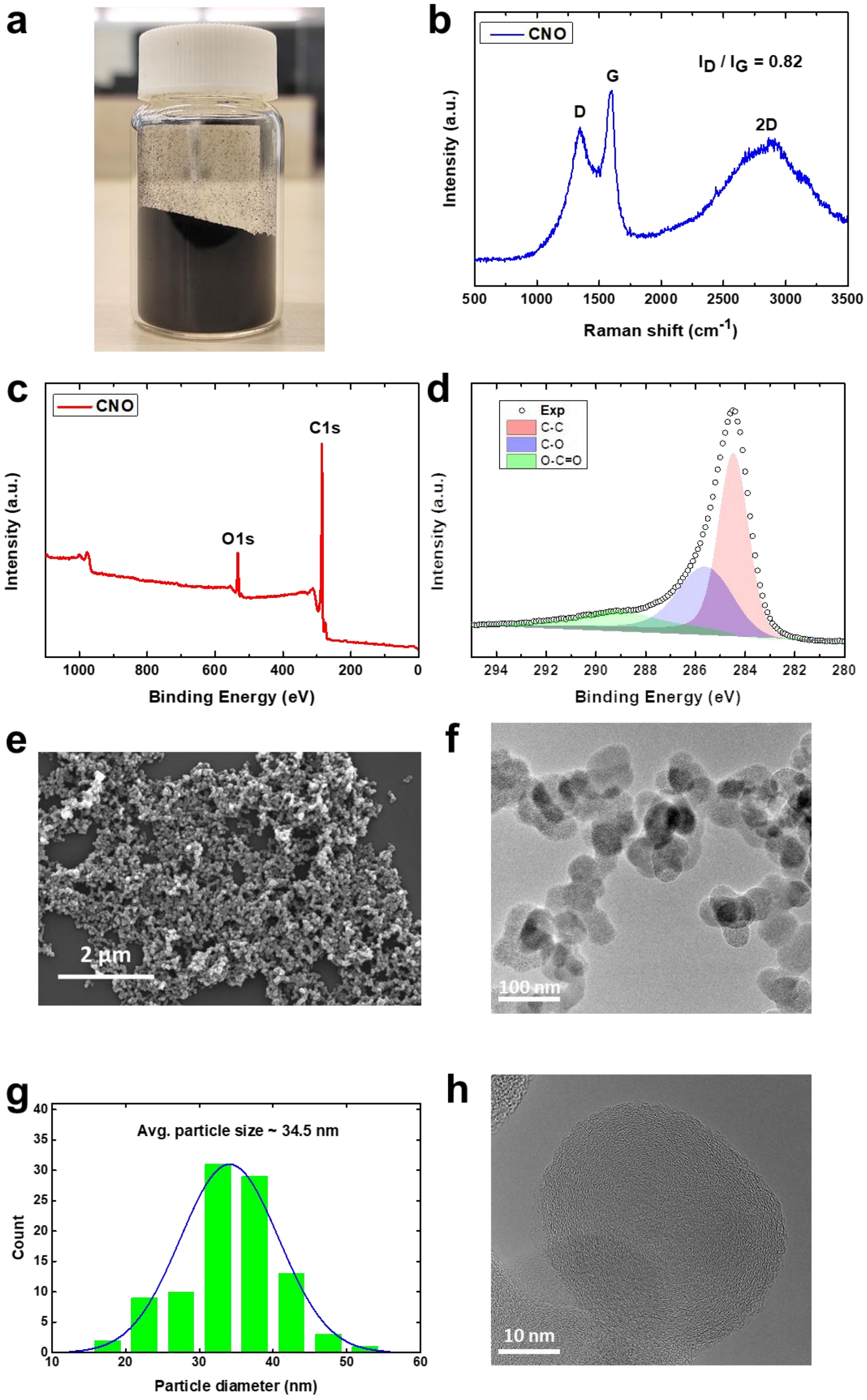

Figure S1. *Physicochemical characterization of the CNOs: (a) photo of the CNO powder; (b) Raman spectrum; (c) XPS survey spectrum and (d) and deconvoluted C1s spectrum. Representative (e) SEM and (f) TEM micrographs images of the CNO particles, highlighting their regular spherical shape and narrow size distribution. (g) Statistical analysis of the particle diameter from the TEM analysis, properly fitted by a Gaussian distribution peaked at ~34 nm. (h) HRTEM image of a single CNO, showing the concentric graphitic rings composing its structure.*

Figure S1a shows a photo of the prepared CNOs in a glass vial. A Raman examination was conducted to study the physical properties of the CNOs. Figure S1b shows the typical Raman spectra of the CNOs. Two characteristics peaks are evident at 1344 cm^{-1} and 1593 cm^{-1}, corresponding to the D and G bands of carbon-based materials, respectively. The D band is attributed to disorder and atomic defects in the graphitic layers of the CNO structure. The G band represents the vibrations of carbon atoms (in-plane stretching) inside the sp^2 graphitic layers. The intensity ratio of the D-band to the G-band provides an indication of the average defect density. The value of 0.82 for the CNOs, which is a useful measure of the defect density, is in line with previously reported values. Furthermore, the presence of a pronounced 2D band (2879 cm^{-1}) indicates the existence of stacked concentric graphitic multilayers, which are commonly found in few-layer graphene samples [1–4]. Understanding the surface chemistry of the as-synthesized CNO is essential for identifying any functional groups, given that the CNOs were produced using an open-air synthesis method. The surface chemical bonding and composition of the synthesized CNOs were analysed using XPS. The XPS survey spectrum (Figure S1c) reveals solely the presence of carbon and oxygen. The high-resolution C1s spectrum (Figure S1d) spectrum shows a clear peak that can be deconvoluted into various components characteristic of graphitic material: the prominent peak at 284.4 eV is associated with sp^2 graphitic carbon, whereas the peaks at 285.6 eV and 289.1 eV are linked to oxidized carbon groups. These may correspond to defects or vacancies within the carbon lattice, which is consistent with the findings from Raman analysis. This demonstrates that the as-synthesized CNO is primarily composed of aromatic sp^2 carbon cores with surface-adsorbed hydroxyl and carboxylic functional groups [5]. The morphology of the CNOs was examined by SEM and TEM, which revealed that the particles were spherical in shape with a narrow size distribution (Figures S1e and S1f). A statistical analysis of the CNO size was performed using TEM, where the diameter of over 120 individual particles was measured (Figure S1g). The diameter histogram was analyzed using a Gaussian distribution (blue line), revealing an average value of particle diameter of 34.5 nm. As anticipated, the interior structure of individual CNOs seems to be composed of many concentric graphitic layers (Figure S1h).

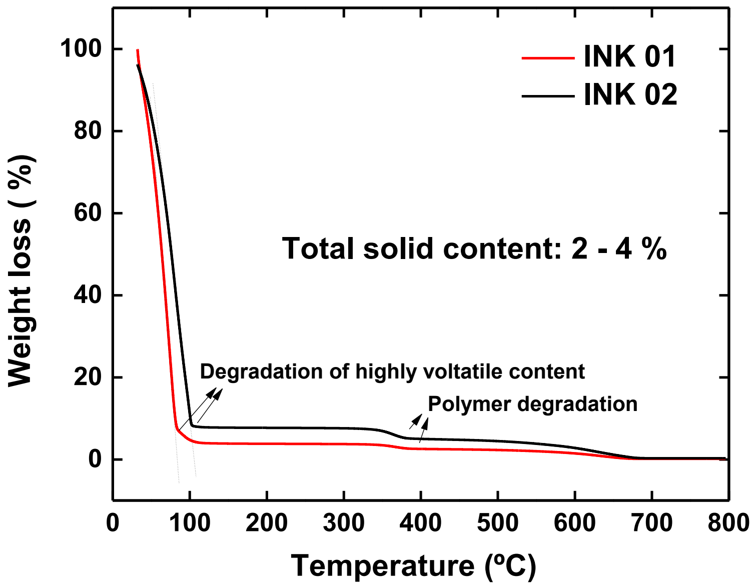

Figure S2. *TGA curves of the* inks in air atmosphere, collected at a heating rate of 10 ºC min^{-1}.

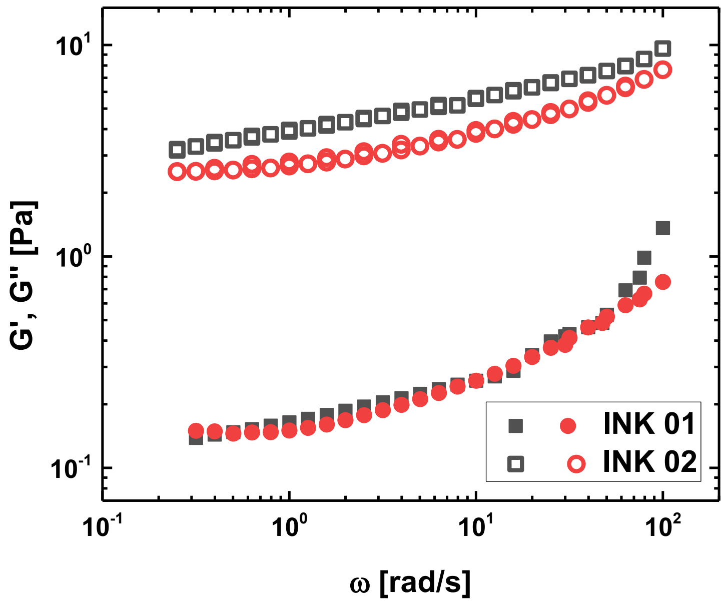

Figure S3. *Dynamic rheological tests. Dependence of the storage modulus (G') and loss modulus (G'') on the angular frequencies in the range of 0.03 - 100 rad·s* $^{-1}$ *at a fixed stress of 10 Pa.*

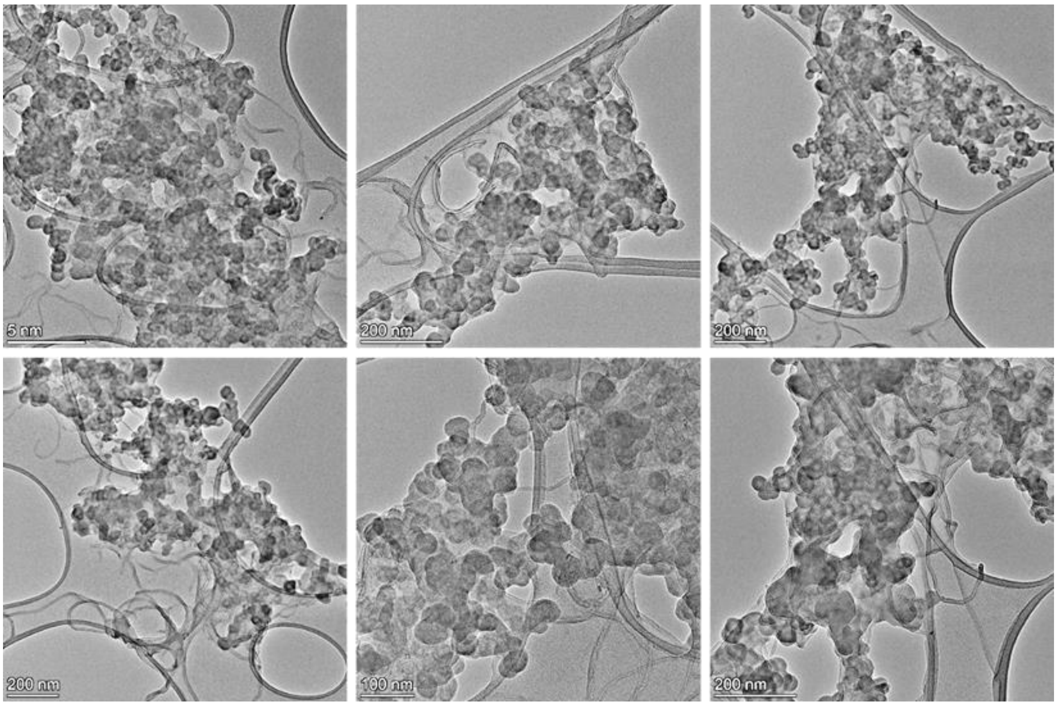

Figure S4: *Supplementary TEM images of the nanocomposite ink.*

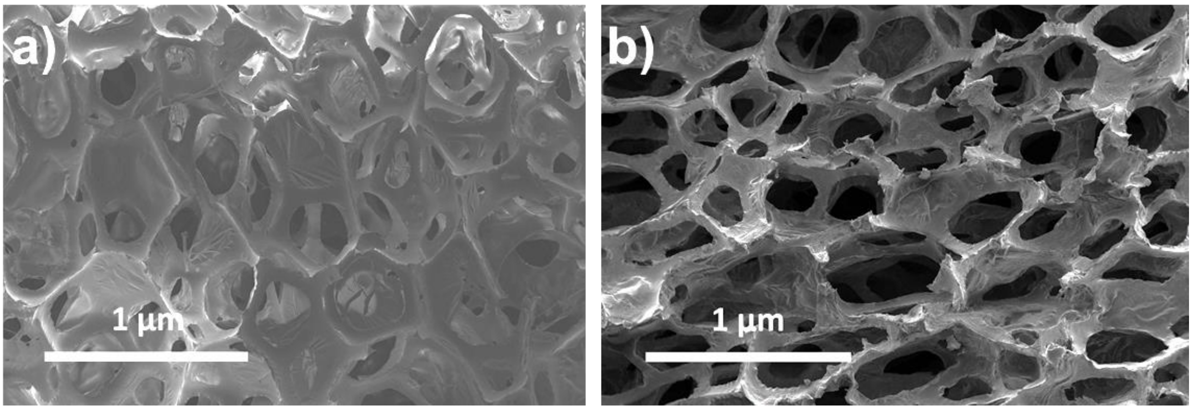

Figure S5: *High resolution SEM images of pristine PU foam (a) and ink coated foam (b)*

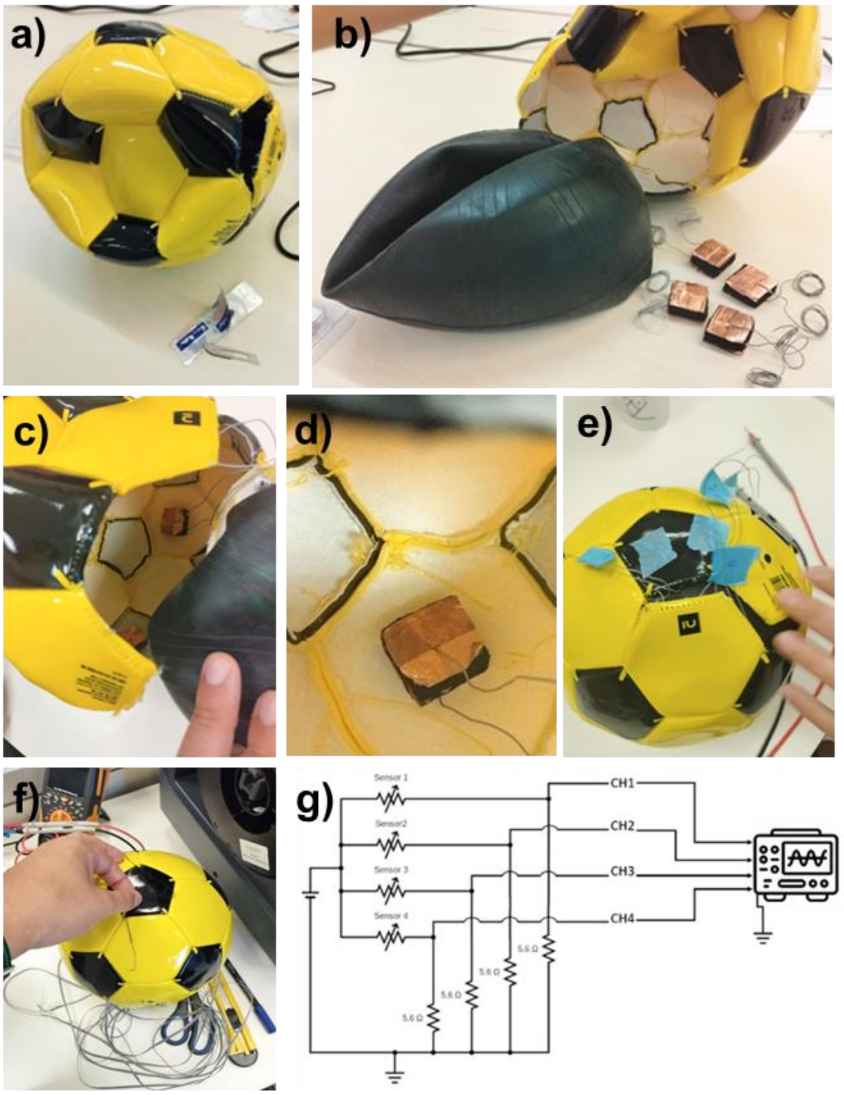

Figure S6: *Football ball sensorization. (a) Partially opened football. (b) Opened football with empty blader and sensors. (c, d) Sensors placed inside the ball. (e) Partially sewed football. (f) Finished football with sensors and needle used during the sewing. (g) Schematics of the readout of the four sensors used in the ball sensing experiment.*

We modified a standard football for sensor integration. The ball was opened by cutting through the surface with a scalpel and then removing the inner bladder. Four compressive strain sensors were fabricated by attaching copper foil and conductive wire to the opposite sides of PU foam blocks (25 mm × 25 mm × 10 mm) coated with composite ink. The connections were secured with conductive adhesive to ensure reliable contact. The four sensors were evenly distributed in a circular arrangement inside the ball and held in place with double-sided tape. The sensor wires were routed towards a single exit point for easy access and connection. Once the sensors were positioned, the bladder was reinserted and partially inflated to provide stability and hold the sensors in place. The wiring was connected according to the circuit shown in Figure S6g, with a common ground and four individual wires allocated for each sensor's measurement. Finally, the ball was accurately sewn back together using a black thread and a hooked needle, and the bladder was fully inflated to playing pressure.

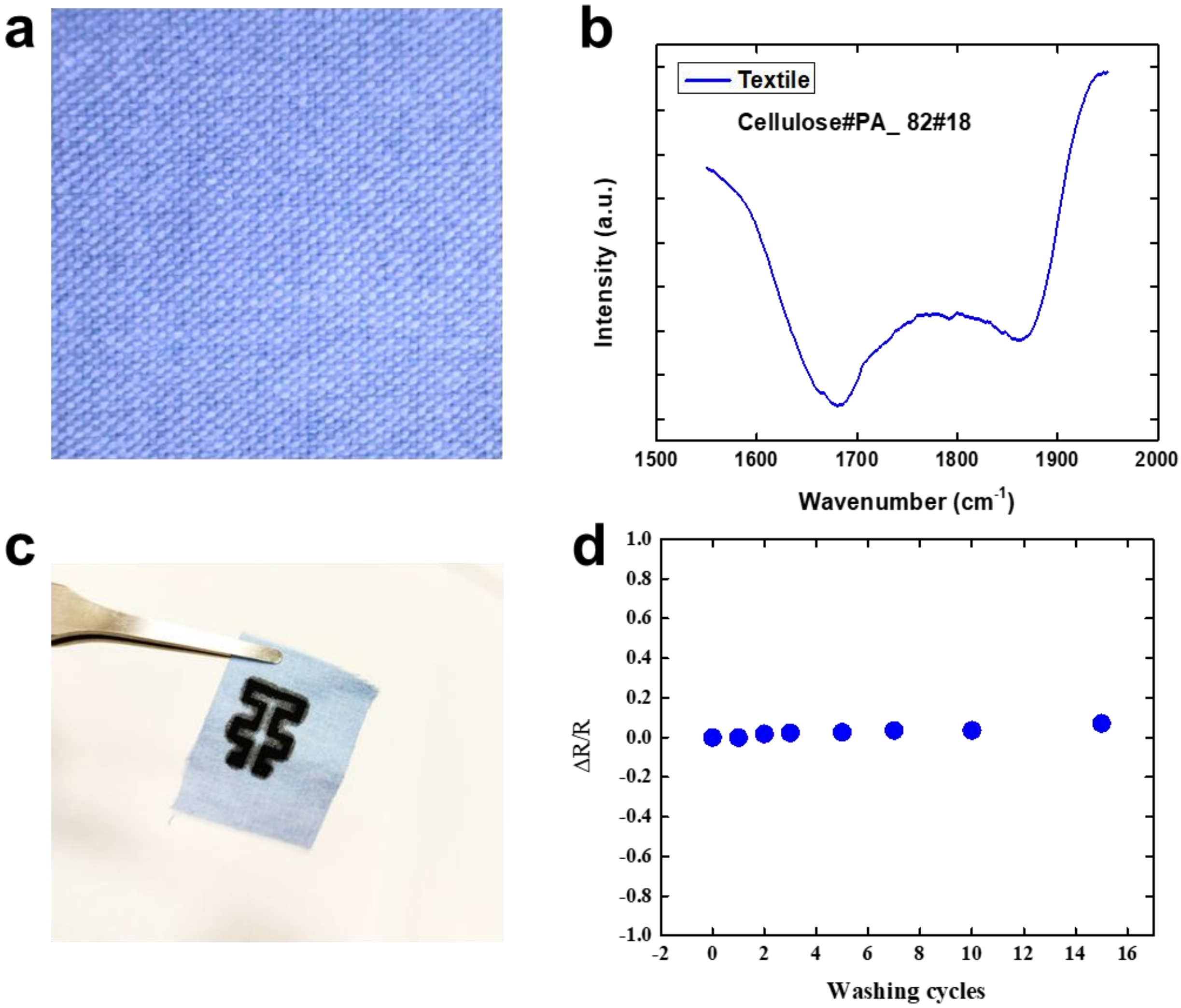

Figure S7. *a) Optical microscope image of the textile b) FTIR spectrum of the textile (containing 82% of cellulose and 18 % of polyamide). c) Photo of strain sensor electrode on the textile d) Stability of the electrode in (c) over washing cycles.*

References

[1] A.C. Ferrari, Raman spectroscopy of graphene and graphite: Disorder, electron-phonon coupling, doping and nonadiabatic effects, Solid State Commun 143 (2007) 47–57. https://doi.org/10.1016/j.ssc.2007.03.052.

[2] M.S. Dresselhaus, A. Jorio, R. Saito, Characterizing graphene, graphite, and carbon nanotubes by Raman spectroscopy, Annu Rev Condens Matter Phys 1 (2010) 89–108. https://doi.org/10.1146/ANNUREV-CONMATPHYS-070909-103919/CITE/REFWORKS.

[3] M.A. Pimenta, G. Dresselhaus, M.S. Dresselhaus, L.G. Cançado, A. Jorio, R. Saito, Studying disorder in graphite-based systems by Raman spectroscopy, Physical Chemistry Chemical Physics 9 (2007) 1276–1291. https://doi.org/10.1039/B613962K.

[4] D. Roy, M. Chhowalla, H. Wang, N. Sano, I. Alexandrou, T.W. Clyne, G.A.J. Amaratunga, Characterisation of carbon nano-onions using Raman spectroscopy, Chem Phys Lett 373 (2003) 52–56. https://doi.org/10.1016/S0009-2614(03)00523-2.

[5] H. Ago, T. Kugler, F. Cacialli, W.R. Salaneck, M.S.P. Shaffer, A.H. Windle, R.H. Friend, Work Functions and Surface Functional Groups of Multiwall Carbon Nanotubes, Journal of Physical Chemistry B 103 (1999) 8116–8121. https://doi.org/10.1021/JP991659Y.
